# Rickettsialpox in North Carolina: A Case Report

**DOI:** 10.3201/eid0807.010501

**Published:** 2002-07

**Authors:** Allan Krusell, James A. Comer, Daniel J. Sexton

**Affiliations:** * Northeast Medical Center, Concord, North Carolina, USA; Centers for Disease Control and Prevention, Atlanta, Georgia, USA; ‡ Duke University Medical Center, Durham, North Carolina, USA

**Keywords:** rickettsialpox, *Richettsia akari*, North Carolina

## Abstract

We report a case of rickettsialpox from North Carolina confirmed by serologic testing. To our knowledge, this case is the first to be reported from this region of the United States. Including rickettsialpox in the evaluation of patients with eschars or vesicular rashes is likely to extend the recognized geographic distribution of *Rickettsia akari*, the etiologic agent of this disease.

Rickettsialpox is caused by infection with *Rickettsia akari*. Disease in humans was first described in 1946 in residents of apartments clustered in a three-block area in the borough of Queens, New York City *1*. Subsequently, small outbreaks of rickettsialpox were recognized in several U.S. cities, including Boston, Cleveland, Philadelphia, Pittsburgh, and West Hartford *2*. Most cases to date have occurred in large metropolitan areas of the northeastern United States; about half the described cases have occurred in New York City. However, rickettsialpox is likely more common in the United States than suggested by the relatively small number of reported cases during the past 50 years *3*,*4*.

*R*. *akari* is transmitted among house mice (*Mus musculus*) and to humans by the house mouse mite (*Liponyssoides sanguineus*) *2*. Recently, cases of rickettsialpox have been reported in residents of the Ukraine *5* and Croatia *6*. Isolations have also been made from Korean voles in an area where rickettsialpox has not been reported *7*. These data suggest that silent sylvan cycles of *R*. *akari* infection exist and that the organism is more widely distributed than currently appreciated. To our knowledge, rickettsialpox has not previously been reported in patients in the southern United States. We describe a recently diagnosed *R*. *akari* infection in a man who resides in a suburban area of North Carolina.

## Case Report

A 48-year-old man who worked at a golf course was admitted to a North Carolina hospital with fevers, chills, headaches, and a rash. Seven days before admission, he noted discomfort on the back of his right calf. The patient stated that he thought something had bitten him at this site, although he had not seen any insects or ticks. Over the next 2 days, thigh tenderness and a papule developed at the site of the original discomfort. Three days before admission, the papule began to ulcerate, and fevers, chills, headaches, and general malaise were present. Two days before admission, several red macules appeared over the anterior chest. Over the next 24 hours, vesicles appeared near the center of these macules.

The patient had a pet dog and cat and had not traveled outside North Carolina in the 3 months before admission. He reported that he had no known exposures to ticks or recent tick bites. He was unaware of any rodents in his house or any local rodent extermination projects. However, he recalled that a stray cat periodically brought dead mice to the general area where he worked, although he never directly touched them.

**Figure 1:**
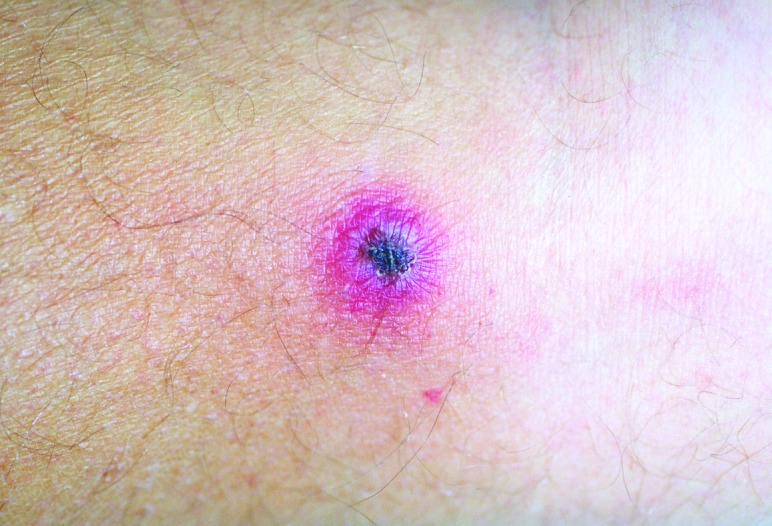
Eschar on posterior right calf of patient with rickettsialpox.

**Figure 2:**
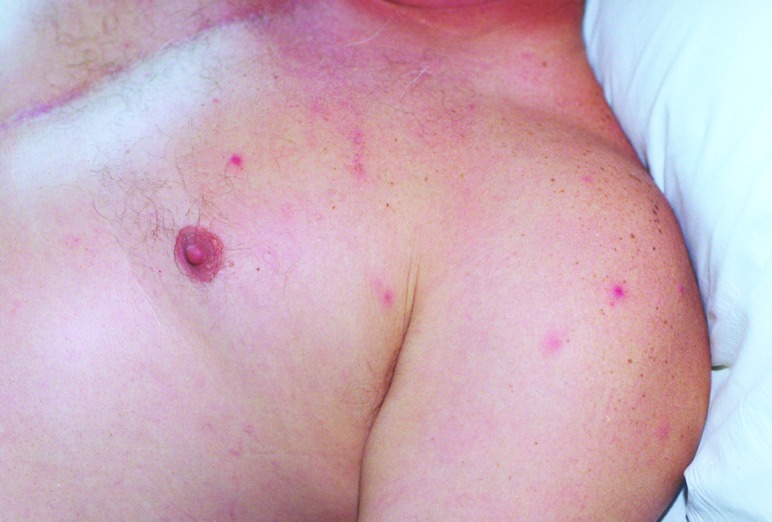
Multiple papulovesicles involving the upper trunk on a patient with rickettsialpox.

**Figure 3:**
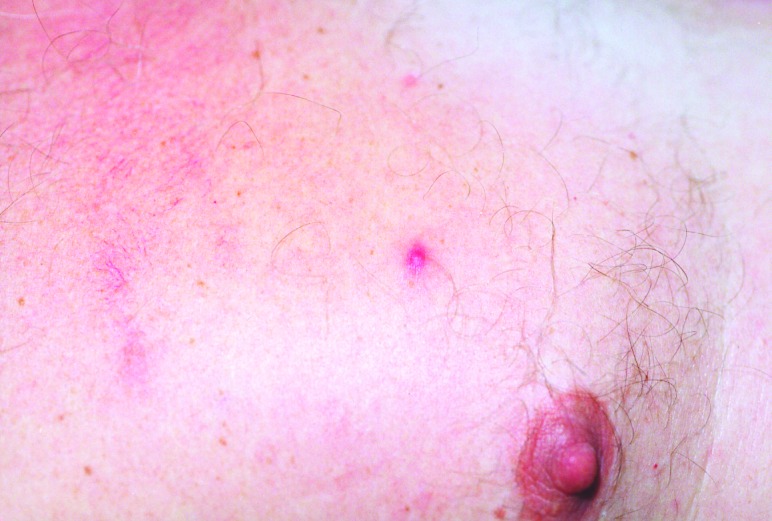
Closer view of papulovesicular lesions on patient with rickettsialpox.

On admission, the patient appeared ill and was febrile. An eschar was present on his posterior right lower leg (Figure 1). Approximately 30 erythematous macules were noted on his trunk, arms, and legs (Figures 2 and 3). Many of these macules had small central vesicles. Laboratory testing showed normal values for electrolytes and creatinine, hematocrit, and leukocytes. His platelet count was 85,000/mm^3^. Routine blood cultures were sterile.

A diagnosis of rickettsialpox was made, and therapy was started with doxycycline and cefazolin. After 48 hours, the patient became afebrile, and his constitutional symptoms abated. He was discharged, took doxycycline orally for an additional 7 days, and recovered completely.

Two serum samples were submitted to the Centers for Disease Control and Prevention for confirmatory testing. Samples were tested by a standard immunofluorescent antibody assay (IFA) for immunoglobulin G antibodies reactive with *R*. *akari* and *R*. *rickettsii* antigens. Because of cross-reactivity among the spotted fever group rickettsiae, confirmatory cross-adsorption testing was done as described *8*. Higher reciprocal titers were obtained to *R*. *akari* antigens than to *R*. *rickettsii* antigens in both samples (reciprocal titers of 1,024 versus 512 on August 29 and 512 versus 256 on October 11, respectively). Adsorption with *R*. *akari* greatly reduced titers to both antigens (<16 to both antigens for both samples), whereas adsorption with *R*. *rickettsii* only partially lowered titers to both antigens (512 versus 256 for the first sample; 128 versus 64 for the second sample). This pattern of differential reduction in titers is confirmatory for a serologic diagnosis of rickettsialpox *9*.

## Conclusions

This patient's illness was typical of rickettsialpox. He had a classic eschar, and his vesicular rash, severe headache, and thrombocytopenia are characteristic findings of infection with *R*. *akari*. However, not all patients with rickettsialpox have a vesicular rash. Although isolation of *R*. *akari* was not attempted and a skin biopsy was not performed, the results of the IFA testing confirmed the diagnosis.

Rickettsialpox may have occurred sporadically in North Carolina in the past, but the incidence of this disease in this state is probably extremely low. As Figures 1–3 illustrate, infection with *R*. *akari* produces unusual and characteristic skin abnormalities. Because cases of rickettsialpox may be confused with chickenpox or other viral exanthematous diseases, misdiagnosis may occur when sporadic cases occur in areas where the disease is unknown to local practitioners. The vesicular rash of rickettsialpox may be easily confused with the skin rash seen in patients with chickenpox; however, the presence of one or more eschars at the site(s) of inoculation, the lack of successive crops of vesicles over time, and the presence of thrombocytopenia should lead clinicians to exclude varicella-zoster virus (formal name: *Human herpesvirus 3)* as the etiologic agent.

In large cities, *R*. *akari* is maintained in a cycle that includes the house mouse and its associated mite *2*. Although our patient did not recall direct exposure to rodents, he did recall a stray cat’s bringing mice into the work area. The patient may have been exposed to infected mites in this manner, although he may also have been unknowingly exposed to rodents and their mites at some other location.

Evaluation of patients with eschars or vesicular rashes for rickettsialpox is likely to extend the recognized geographic distribution of *Rickettsia akari*, the etiologic agent of this disease.
